# Strength Classification of Wooden Chairs under Cyclic Loads Based on an Experimental Study

**DOI:** 10.3390/ma16196580

**Published:** 2023-10-06

**Authors:** Harun Diler, Ali Kasal, Tolga Kuşkun, Yusuf Ziya Erdil, Ersan Güray

**Affiliations:** 1 Department of Materials and Material Processing Technology, Vocational School of Technical Science, Akdeniz University, Antalya 07058, Türkiye; hdiler@akdeniz.edu.tr; 2 Department of Wood Science and Industrial Engineering, Faculty of Technology, Muğla Sıtkı Koçman University, Muğla 48000, Türkiye; tolgakuskun@mu.edu.tr (T.K.); erdil@mu.edu.tr (Y.Z.E.); 3 Department of Civil Engineering, Faculty of Engineering, Muğla Sıtkı Koçman University, Muğla 48000, Türkiye; ersan.guray@mu.edu.tr

**Keywords:** acceptable design loads, wooden chair, furniture joints, frame construction

## Abstract

This study aimed to assess the cyclic load capacity of wooden chairs and subsequently categorize them based on their performance. A diverse selection of chair models was randomly procured from commercial markets. These chairs underwent performance testing, utilizing the cyclic stepped increasing loading method, with adherence to the standards set forth by the American Library Association Technology Reports (ALA). The study evaluated 315 chairs, encompassing 21 chair models. Each chair model underwent five replications of testing across three different loading directions. The resulting dataset of numerical values was subjected to statistical analyses, facilitating the categorization of chairs based on their strength under cyclic loads. Notably, the study revealed substantial variations in the load capacity among different chair models. As a consequence of this investigation, the study established acceptable design load thresholds. For instance, concerning front-to-back loading, it was determined that the chairs with cyclic load capacities ranging from 932 to 1449 N fell within the category of low-strength, between 1450 and 1968 N were classified as medium-strength (suitable for domestic use), and the chairs with cyclic load capacities exceeding 1968 N were considered to possess high strength (intended for hotel lobbies, restaurants, libraries, etc.). Similarly, for back-to-front loading performance, the study identified the chairs with cyclic load capacities between 625 and 895 N as low-strength, 896 and 1167 N as medium-strength, and the chairs with loads surpassing 1168 N as high-strength. The performance thresholds for side thrust loads were as follows: low-strength encompassed the cyclic load capacities ranging from 649 to 934 N, medium-strength spanned the cyclic load capacities between 935 and 1221 N, and high-strength entailed 1222 N and above. Notably, the classification devised in this study is closely aligned with the widely accepted and internationally recognized ALA specification. This strong consistency with global standards reinforces the reliability and applicability of the classification system developed in this research. In conclusion, this study enhances understanding of wooden chair strength performance and offers practical insights that lead to higher-quality products and improved consumer satisfaction. Its recommendations can potentially drive positive change within the industry and benefit manufacturers and consumers.

## 1. Introduction

Furniture could be described as the most important product in human life in every area of everyday life, directly affecting the level of comfort of the individual and society, responding to social and cultural needs [1]. A total of 99.2% of furniture users renovate their furniture within approximately 15 years. In addition, 85% of users are renovating their furniture over a period of about 3–10 years, and the renovation process is expected to take place at shorter intervals in the future [2,3].

Türkiye’s share in world furniture exports in 2016 was 1.1 percent; in 2022 it was 2.1 percent [4]. This shows that Türkiye’s furniture exports have increased gradually, and it is important to determine the service loads for exports of furniture to European countries.

The furniture industry is one of the oldest and most developing sectors in Türkiye. In recent years, facilities that produce world-class products have been established in the sector, and a position has been reached in which it sells products throughout the country and the world through dealership organizations. The sector improves its products and increases its diversity every year. The sector aims to reach 25 billion dollars of production and 10 billion dollars of exports in 2023 and to be among the world’s top 10 and Europe’s top 5 largest furniture manufacturers [4].

In Türkiye, furniture manufacturing companies rarely use furniture testing techniques. In fact, although there are test standards compatible with European standards to determine the performance of all types of furniture [5], the application of these tests is not common. Because of this, furniture companies do not have to comply with the relevant national and/or international furniture standards.

Considering the reflection of the standards in the furniture industry of Türkiye, according to the International standard code list (ICS), “Wood Technology” is classified as 79 and “Furniture” is classified as 97.140. Furniture standards are prepared by furniture technical committee (CEN/TC 207) and international furniture technical committee (ISO/TC 136) and are grouped as home furniture, office, outdoor, and educational furniture [6].

The test standards and loading methods used to determine the strength and durability of chairs in Türkiye do not fully represent the real usage conditions. In the applied tests, the fact that the members and joints that construct the chair system are subjected to tests with only static or simple fatigue loading, without being exposed to the fatigue effect due to time and variable repetitive loading, ensures that the chair shows the highest strength value and can easily meet the static or simple fatigue test loads. However, in real use, the members and joints that construct the chair system undergo serious deformations at lower levels than the loads obtained in the tests and assumed to be able to carry, due to the effect of time and the repetition and variability in loading. Another aspect of the current tests is that chairs are subjected to pass/fail tests, and if they exceed a certain loading, they are considered to have passed the test successfully. However, in this type of test, chairs that pass the test with load values slightly above the loads accepted as the strength limit and chairs that pass the test with load values much above are evaluated in the same category. In other words, the life time of the chairs is not determined, the test is completed as soon as the acceptable load is exceeded. This situation results in chairs with greater strength than the loads they must carry, especially during use, and creates high costs for manufacturers.

In the furniture industry, product engineering methodology, which is a natural and crucial part of furniture design, is not yet systematically implemented in the world. As a result, many items of furniture cannot perform their functions properly during usage and become unusable in a short period of time, as they do not have sufficient strength value. Some products are manufactured with a strength that can carry much more of the potential loads they may be subjected to during use, and this leads to both economic and aesthetic issues [3].

Furniture structures are in the field of product engineering, and have been exposed various intensities of loads. Thus, the engineering design of furniture has be to applied systematically during the manufacturing process. Furniture structures should meet the strength and durability requirements. Furniture structures fail very often from the joints, which are the critical and weak points of the whole system. Performance tests of furniture have an important role in evaluating and estimating strength and durability during its service life [7].

Performance tests are the last phase of the furniture engineering methodology. The aim of these tests is to recognize the strength weaknesses that may arise during usage and to provide the furniture designer with information about the furniture’s strength in order to make appropriate improvements before it is in use and manufactured. As a result, furniture performance tests could be described as simulations used in the engineering design process to see whether the required functions of the furniture are satisfied or not [8,9].

In many of European countries, no works have been performed for determining the acceptable design values regarding the loads necessary to carry by furniture, specially the chair frames. The lack of a related database and the failure to implement the performance testing methods and furniture engineering in research and development (R&D) departments of the companies result in the appearance of products that display very different strength performances, even though they are manufactured to serve the same duty.

Cyclic stepped increasing loading appears to be best suited for utilization in performance tests of any kind of furniture. This loading method includes an interaction between “initial load”, “load increment”, “load cycles at each load level”, and “total cycles” [8,10]. In the methodology of this loading, a specified load is applied to the furniture at a specified cyclic rate for a specified number of cycles. After the arranged number of cycles is finalized, the load is increased by a given increment and the process is repeated. This process is continued until a desired load level has been reached, or until the furniture fails [8]. There are many studies in the literature where furniture performance tests have been performed using this loading method.

Some studies by Eckelman and Haviarova compared the strengths of school chairs made with glued but unpinned round mortise and tenon joints to the strength of chairs with glued but unpinned joints. According to the findings, round mortise and tenon joints with small cross pins are an alternative method of joint construction when adhesives are in short supply. These joints offer nearly the same strength and durability as equivalent chairs made with glued joints [11].

It was discovered that domestic chairs manufactured in Türkiye to fulfill the same purpose have a wide range of strength performance variations and a clear pattern of inconsistency between manufacturers and models emerged. The lack of an R&D culture among manufacturers or a lack of awareness of or non-implementation of performance testing techniques is thought to be the cause of this kind of situation [12].

The relations between the dimensions of the furniture and a user’s anthropometric data is crucial for ergonomics, safety and functionality. The weight and dimensions of the user’s body significantly affect the functional dimensions of the furniture, especially for overweight users [13,14]. It was stated that obesity, which has been a global epidemic in recent years, has significant effects on furniture design, especially in public spaces, and a study was conducted on the strength of chairs used by people with higher body mass [15].

Furniture frames consist of members and the systems called joints, which are formed by connecting these members to each other at their ends. The strength of the whole furniture is represented by the strength of the members and joints. In order for the furniture to safely carry the loads it may be exposed to during use, both its members should be strong and the joints should have sufficient strength. It is clearly understood from the failure modes observed in past performance tests that joints are critical points in furniture constructions. Therefore, the strongest joints should be used in the furniture frames, which are their most important components. However, the cross-sectional properties (dimension, geometry, and orientation) of the members that make up the system are also effective on the strength of entire system. In order to carry the service loads and apply the joints, the cross-sectional dimensions of the members that forming the system should be large enough. Additionally, corner support elements, metal brackets, etc., are also used to increase the strength of the system.

For thousands of years, woodworkers have utilized mortise and tenon joints to connect the pieces of hardwood parts, typically when adjacent pieces connect at a 90° angle. They continue to be preferred to construct furniture frames. Traditional mortise and tenon joints are also commonly used in the leg joints of chairs [16,17,18,19]. The length, depth, and thickness of the tenons, the type of fit, the geometry of the plug and hole, the thickness of the glue line, the wood species used, and the adhesives used are just a few of the numerous variables that determine the bending moment capacity of mortise and tenon connections [20,21,22]. The joints of wooden chairs are frequently subjected to internal and external stresses during usage [23]. As a result, it is essential to consider the design of the chair’s connecting joints [24,25,26,27,28]. According to Eckelman and Haviarova [11], failure or damage to the connecting joint is a common cause of damage to wooden chairs. Derikvand and Eckelman (2015) investigated the moment resistance of end-to-side floating tenon joints as a function of tenon shape, geometry of the tenon surfaces, bond line thickness, tenon width, and wood species. According to the findings, loose tenons with round edges were 20% more powerful than those with rectangular edges that were seated into round-end mortises. The degree of fit between the tenon and the inside walls of the mortise had the biggest impact on how much the joints could bend. The beech joints with 0.05 mm thick glue lines and 45 mm wide grooved tenons had the maximum bending moment capacity [29].

There are a few investigations and no established standards for acceptable design loads for industrially produced wooden chairs. Because of this, manufacturers are unsure of the strength of the chairs they manufacture. In another words, depending on the manner of usage, it is unclear whether the manufactured chairs will be appropriate for the intended use or whether they can withstand potential loads. The primary goal of this study is to classify chair models according to strength, which will lead to the identification of acceptable design loads for low-, medium-, and high-strength groups for the manufactured chairs. The study uses wooden chair models constructed from Turkish beech wood for domestic use by furniture industry companies.

## 2. Materials and Methods

### 2.1. Chair Models Used in the Study

Strength properties of 21 various household chair models that were acquired from companies in three separate cities (Ankara, Bursa, and Kayseri) in Türkiye, where the furniture industry is at the forefront. Data were collected after 315 tests were conducted using 21 different chair types, 3 loading methods, and 5 replications of each chair model.

The extensive distribution of Turkish beech wood (*Fagus orientalis* L.) in Türkiye and its widespread use in the furniture industry are the reasons that all of the chosen chairs were made from this premium material.

**Figure 1:**
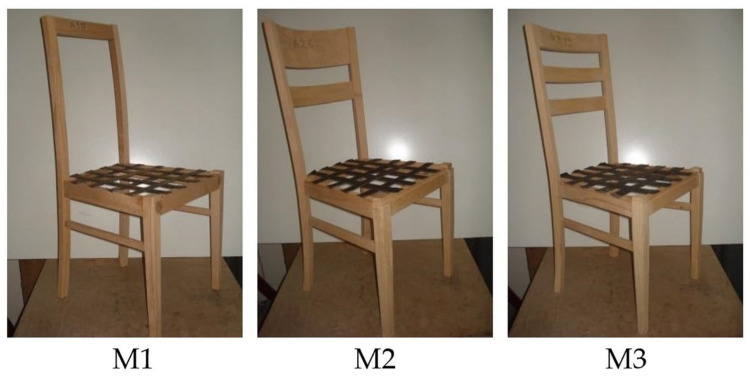

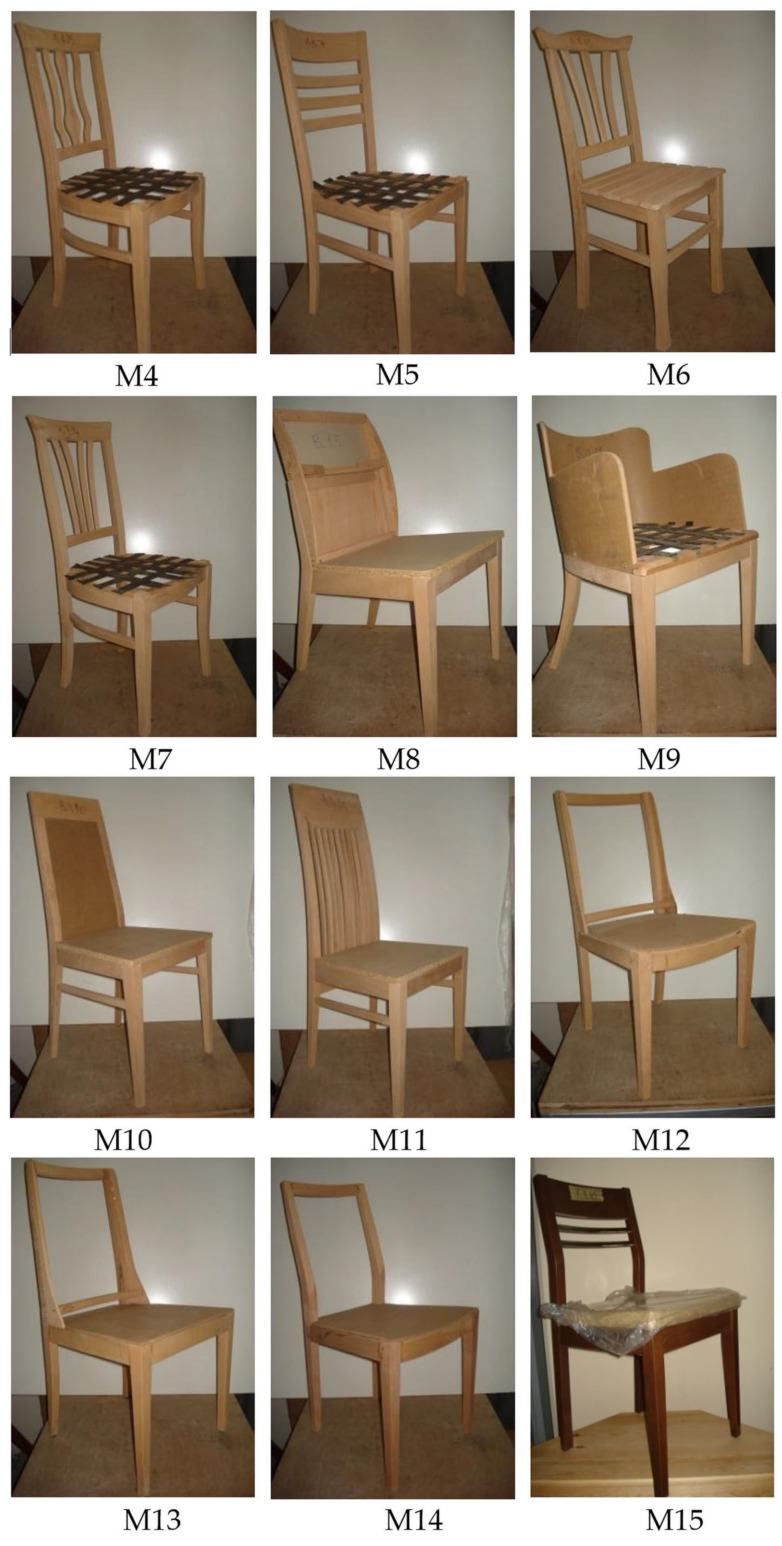

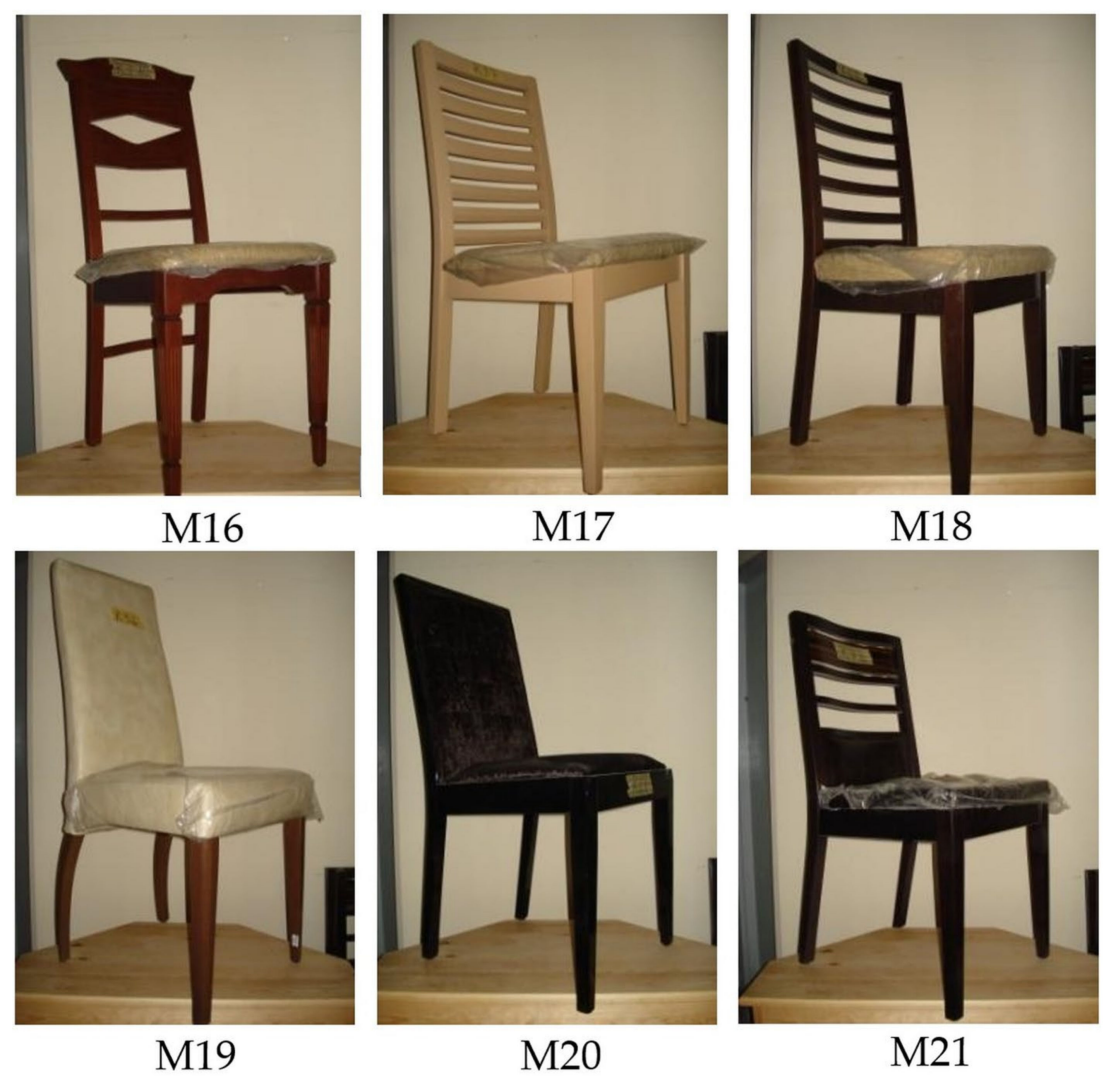
The chairs (M1–M21) evaluated within the scope of the study.

The photos of each model are shown in Figure 1, together with the total number of chair types (21), which were chosen as 7 models from each of the cities.

From the market, chairs were selected entirely at random. All chairs were supplied as finished products. However, some of the chairs supplied were not painted or varnished and were not upholstered (M1–M14). Although the painting or varnishing process did not have a significant effect on the strength, in order to reflect the effect of the upholstery process on the strength in the performance tests, elastic webbing supports were stapled to the un-upholstered chairs to represent the effect of the upholstery on the strength (M1, M2, M3, M4, M5, M7, and M9).

A polyvinyl acetate (PVAc) adhesive was utilized at the joints to assembling the 14 of the chairs (M1–M14). Seven chair models provided were ready to assemble (RTA), and mechanical joints (socket screws) were utilized where necessary (M15–M21).

### 2.2. Performance Tests and Loading Used in the Study

**Figure 2:**
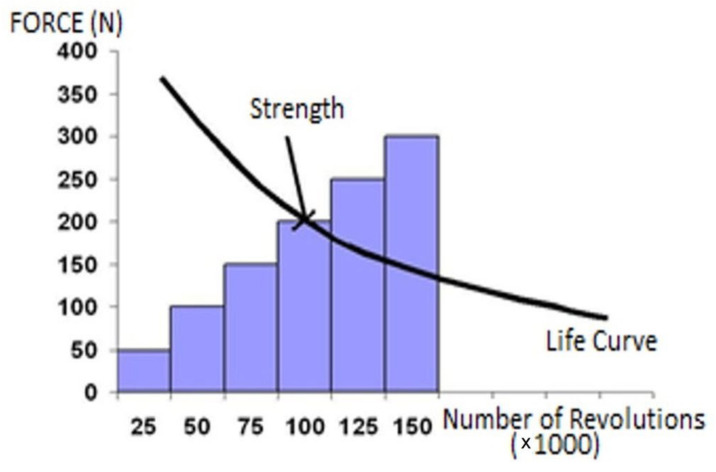
The cyclic stepped increasing loading methodology.

In the study, the “cyclic stepped increasing method”, which simulates actual user loading activities, was utilized in the performance tests. By identifying the initial crossing point of a product’s life curve and the influences of external forces, this method efficiently replicates the performance that any product can struggle against the potential problems met during their service lives (Figure 2) [10,30].

In the tests, chairs were loaded using the cyclic stepped increasing loading method at a specific rate and speed for each performance test. After completing this phase, the load amount was increased once again within a preset range to repeat the first step. These procedures were carried out repeatedly until the acceptable design load values were obtained or the furniture displayed failure, such as openings or breaks, etc. The speed was 20 cycle per minute, and there were 25,000 cycles every step. By comparing this performance value to acceptable design load values defined in the standards for light, medium, and heavy usage, required optimizations can be made. This performance value was then tested for durability. In this approach to testing, “light service” represented the use of private and domestic spaces, “medium service” represented the use of office places which are not very intensive, and “heavy service” represented the use of institutional places such as hospitals, schools, libraries, and restaurants [10,30].

### 2.3. Front-to-Back Load Capacity Test

This test method includes pulling the chair seat frame system from the front to the back while sustaining this loading until the chair permanently deforms, the joints begin to open or and members break, etc. This test aims to establish the doubling strength of the side frame joints. According to the failure modes of this test, the appropriateness of the application of glue, its quantity, and the accuracy of the joint constructions used in the side frame joints could be evaluated. This kind of loading simulates the process of leaning back when seated in a chair [9].

**Figure 3:**
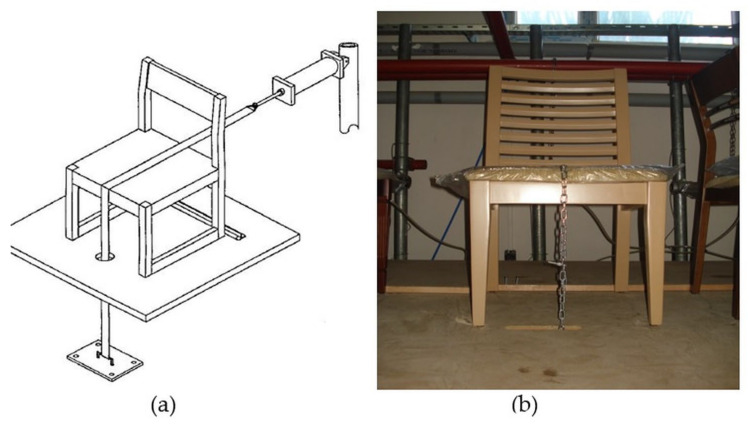
(**a**) Test setup for front-to-back loading; (**b**) applied tests to chairs in the study.

In the tests, front-to-back loading was performed at 20 cycles per minute (Figure 3a,b). The experiments began with a load of 445 N, and continued with an increase of 112 N after each successful completion of 25,000 cycles. After each completed load step of 1113 N, the load increase value was increased from 112 N to 224 N. Loading was continued until the chair had completed its life time, As stated for the previous performance test [9,30].

To prevent the test chair from sliding backward, support pieces were positioned on the back of the chair’s back legs, as indicated in the Figure 3a,b. With the aid of a chain connected to the piston that applied the tensile force, front-to-back loading was made possible. The loading chain was situated in the center of the chair’s depth direction. During the testing, it was found that the loadings had broken in the chair members, the joints had been opened up, and so on [9,10,30].

### 2.4. Back-to-Front Load Capacity Test

Using this test method, the chair seat frame system is forced from the back to the front, and this loading is applied until the chair permanently deforms, the joints open, and the members break. Similar to the front-to-back loading test, the aim of this test is to assess the joint strength in the side frames of chair. It is a test that helps to determine whether to use glue, how much glue to use, and whether the joints on the side frame are constructed in accordance with method are all appropriate when the loading direction is changed [9,10,30].

**Figure 4:**
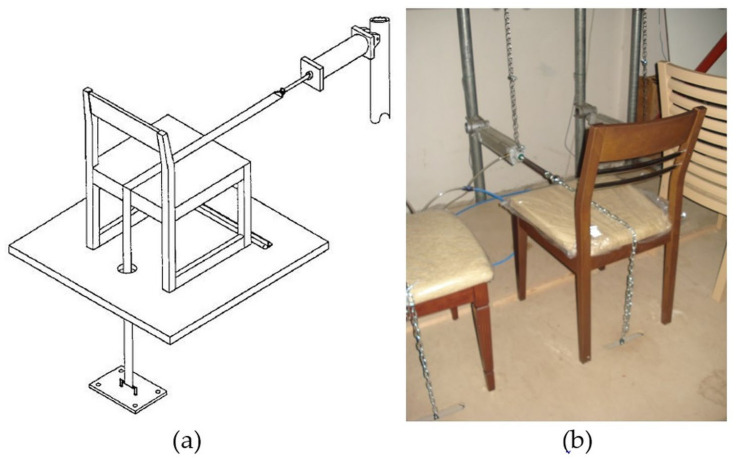
(**a**) Test setup for back-to-front loading; (**b**) tests applied to chairs in the study.

Tests were carried out with back-to-front loading of 20 cycles per minute (Figure 4a,b). Tests began with a load of 445 N, with each successful completion of 25,000 cycles continuing with an increase of 112 N. After each completed load step of 1001 N, the load increase value was increased from 112 N to 224 N. Loading continued until the chair completed its lifetime, as with the previous performance tests [9,10,30].

To stop the test chair from sliding forward, support pieces were positioned on the front bottoms of the front legs, as seen in the Figure 4a,b. A chain that was locked to the piston and attached to the piston that applied the tensile force allowed for back-to-front loading, and the loading chain was situated in the middle of the chair’s breadth direction. In the studies, the loads were raised until the chair’s members underwent extreme deformation, such as breaking or opening joints, and lost their ability to support weight. The chair’s life was then calculated based on the number of cycles and load value at the time the chair failed [9,10,30].

### 2.5. Sidethrust Load Capacity Test

The chair seat frame system is driven laterally during this test procedure, and the loading continues until the chair permanently deforms, joints open, and elements fail. The major goal of this test method is to evaluate how well the chair performs when subjected to sidethrust coercive forces. Such loadings occur when doing things like lying down, bending the chair sideways for any reason, or pushing the chair laterally by leaning against the armrest, especially when speaking to someone who is lying on their side. This test’s objective is to evaluate the joint strength holding the side frames together [9,10,30].

**Figure 5:**
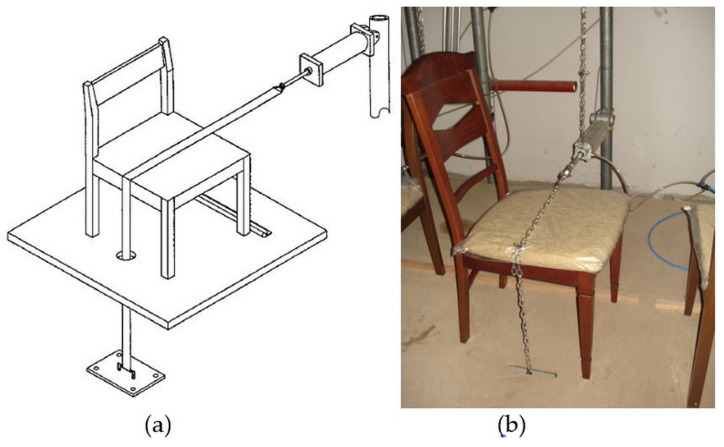
(**a**) Test setup for sidethrust loading; (**b**) tests applied to chairs in the study.

In the tests, sidethrust test was carried out at 20 cycles per minute (Figure 5a,b). The tests began with a load of 223 N, and continued with an increase of 112 N after each successful completion of 25,000 cycles. After each completed load value of 1113 N, the load increase value was increased from 112 N to 224 N. Loading continued until the chair completed its lifetime, as with the previous performance tests [9,10,30].

To stop the test chair from sliding sideways, support pieces were positioned on the bottom part of the chair, as seen in the Figure 5a,b. With the help of a chain connected to the piston that applied the tensile load, sidethrust loading was made possible. The loading chain was situated in the depth direction’s center of the chair and was connected to the piston. In the studies, the loads were raised until the chair elements underwent extreme deformation, such as breaking or opening joints, and lost their ability to support weight. The load value and rotations until the chair broke were recorded as its properties [30].

### 2.6. Strength Classification of the Chairs

The strength values for each loading in this investigation were categorized. A classification study was carried out for the sample group chosen to represent Türkiye, consisting of 21 types and 5 replications from each type, for a total of 105 chairs’ performance values tested for each groups of loading type. In the scope of the study, a total of 315 chairs were tested and evaluated.

According to this method, the mean of the data obtained for each loading direction was distributed as a normal distribution, and 34% of the data that contain a standard deviation (SD) and fall below the mean value were deemed to be “weak strength,” while 34% of the data that contain a SD and fall above the mean value were deemed to be “medium strength”. A group of 14% was considered “inadequate”, and 14% was considered “high strength”, since they were out of the target range. In this classification; “inadequate” refers to the chairs that are not suitable for domestic use, “weak strength” refers to the chairs that may be suitable for domestic use if improvements are made in some aspects, “medium strength” refers to the chairs that are suitable for domestic use, and “high strength” refers to chairs that are for use more intensive than domestic use (for hotel lobbies, restaurants, libraries, etc.).

**Figure 6:**
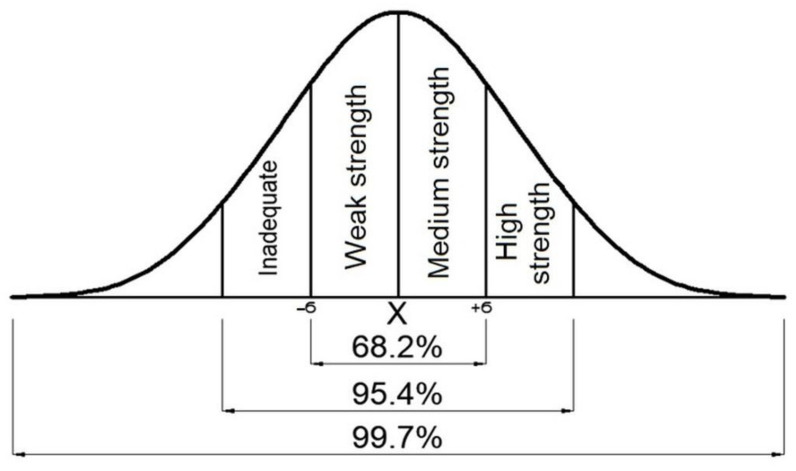
Regular distribution and classification.

As expected at this point, the data for each loading group exhibit a regular distribution (Figure 6).

It was confirmed from the results of the “Single Sample K-S (Kolmogorov–Smirnov) test” that the sample data of the front-to-back, back-to-front, and sidethrust loading groups were consistent with the defined population normal probability distribution (Table 1).

According to the results of Single Sample K-S (Kolmogorov–Smirnov) test, it can be said that the data obtained for each loading show “normal distribution”. Accordingly, after this stage, the classification stage was identified for each loading direction.

## 3. Results and Discussion

### 3.1. Some Physical and Mechanical Properties of Wooden Materials

The physical and mechanical properties of the wood used in the production of chairs are given with the coefficients of variation in Table 2.

### 3.2. Classification Results for the Front-to-Back Load Capacity Tests

In the classification of front-to-back load capacity test results, the results of the statistics according to the method that the data are considered to be normal distribution are given in Table 3.

According to Table 3, the mean of the group was 1449.8 N and the SD was 517.7. The COV of the group was 35.7%. The high COV indicates the inconsistency between the cyclic front-to-back load capacities of chair models.

For the medium-strength group of chair models, the lower limit was calculated, using these data, to be 1450 N; the mean value for the classification and the upper limit was determined to be 1968 N, which was above the standard deviation. In another words, chair types which were within the 1450 to 1968 N range for front-to-back load capacity values were considered to be in the “medium strength” category and considered suitable for domestic usage. For inadequate-strength chair models, the 932 N value, which is less than the mean value, was the absolute minimum. As a result, chair models with front-to-back load capacity values between 932 and 1449 N are regarded as “weak strength”, and require improvements to increase the strength. The highest load capacity limit for medium-strength chairs is 1968 N, and chair types that perform above this (high-strength) can be considered suitable for usage, as well as for heavier services like in libraries and restaurants. The classification of the chair models is given in Table 4.

**Figure 7:**
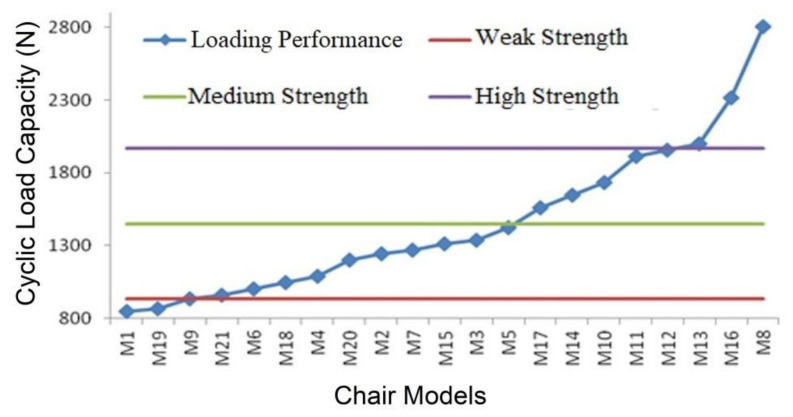
Cyclic load capacities under front-to-back direction for chair models.

The evaluation of each chair types for the classification performed for front-to-back load capacity is presented in Figure 7.

As seen in Figure 7, even though 13 of the 21 chair models tested (62%) were below medium-strength and were manufactured for domestic usage; they were considered unable to satisfy the usage requirements. A total of 2 chair models (M1 and M19) were classified as having insufficient strength, 11 (M9, M21, M6, M18, M4, M20, M2, M7, M15, M3, and M5) as having low strength, 5 (M17, M14, M10, M11, and M12) as having medium strength, and 3 (M13, M16, and M8) as having high strength. It may be said that the 3 high-strength chairs are substantially stronger than needed for domestic usage, while the 13 remaining models in the medium-strength category still need serious strength improvement adjustments. In this group, there were only five varieties that could be used domestically under front-to-back loading conditions. Technical issues with inadequate examples of other types include the requirement for strength enhancements or models with exceptional strength that have aesthetic and economic issues. Engineering design methodology should be used to resolve these issues.

### 3.3. Classification Results for the Back-to-Front Load Capacity Tests

Table 5 indicates that the statistics of classification for back-to-front load capacity test results of the chair models.

Accordingly, the group mean was calculated as 895.9 N and the SD was 271.2. The COV of the group is 30.2%. The high COV demonstrates the inconsistency of the cyclic back-to-front load capacities of chairs.

These data were used to identify the lower limit value for the medium-strength of chairs as 896 N, the mean value for the categorization, and the maximum limit as 1167 N, which was above the SD. In another words, “medium strength” groups of chair types with load capacities within the range of 896 to 1167 N for back-to-front load capacity values were allowed and assessed as suitable for domestic usage. The lower limit for weak-strength chairs was the 625 N value, which was less than the mean value. As a result, chair models with back-to-front performance values between 625 and 895 N are regarded as “weak strength” groupings, and were thought to require modifications for strength development. It was simple to consider chair models that perform over the maximum limit of medium-strength chairs, 1167 N, to be ideal models for domestic usage, as well as heavy services like libraries and restaurants.

Table 6 present the classification obtained based on the back-to-front load capacities of the chair models.

**Figure 8:**
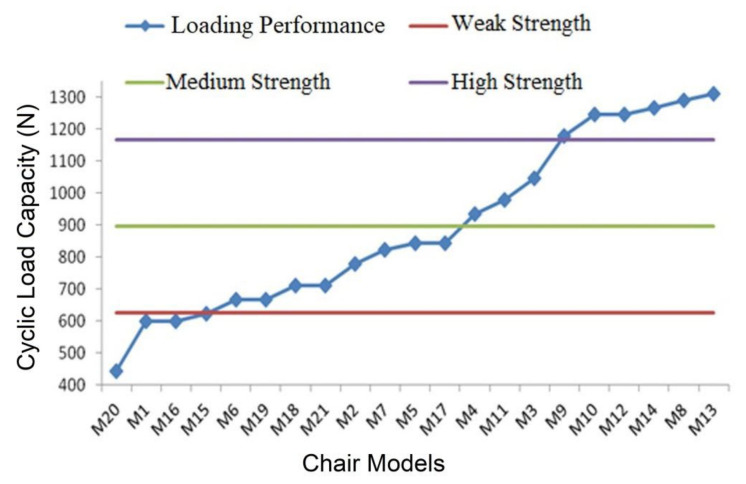
Cyclic load capacities from back-to-front direction for chair models.

The evaluation of each chair types for the classification performed for back-to-front load capacity is presented in Figure 8.

In accordance with this, 12 chairs out of the 21 that were evaluated, or 57%, fell below the medium strength and, while being made for domestic usage, were deemed incapable of meeting the usage requirements. According to the classification results, 4 models (M20, M1, M16, and M15) were defined as inadequate-strength, 8 (M6, M19, M18, M21, M2, M7, M5, and M17) as weak-strength, 3 (M4, M11, and M3) as medium-strength, and 6 (M9, M10, M12, M14, M8, and M13) as high-strength. It may be noted that the 6 high-strength chairs are substantially stronger than required for domestic usage, while the 12 models still in the medium-strength category need serious strength improvement optimizations. In this group, there were only three models that were acceptable for domestic usage under back-to-front loading.

### 3.4. Classification Results for the Sidethrust Load Capacity Tests

Table 7 shows that the statistics of classification for sidethrust load capacity test results of the chair models.

As a result, a group mean of 935.1 N and a SD of 285.7 were determined. The group’s COV is 30.5%. The high COV demonstrates the inconsistency of the cyclic sidethrust load capacities of chairs.

These data were used to identify the lower limit for the medium-strength chairs to be 935 N, the mean value for the classification, and the upper limit to be 1221 N, which was above the SD. Accordingly, chair types were recognized as “medium strength” groups and assessed as suitable for domestic usage if their sidethrust load capacity values ranged from 935 to 1221 N. The lower limit for weak-strength chair models was 649 N, which was less than the mean value. As a result, chair models with sidethrust load capacities between 649 and 934 N were determined to be “weak strength” categories and in need of improvements for strength development. Chair types that have strength above 1221 N, which is the upper limit of medium-strength chairs, could be considered as models suitable for heavy usage, such as in libraries and restaurants.

The classification results for the sidethrust load capacity of the chair models are presented in Table 8.

**Figure 9:**
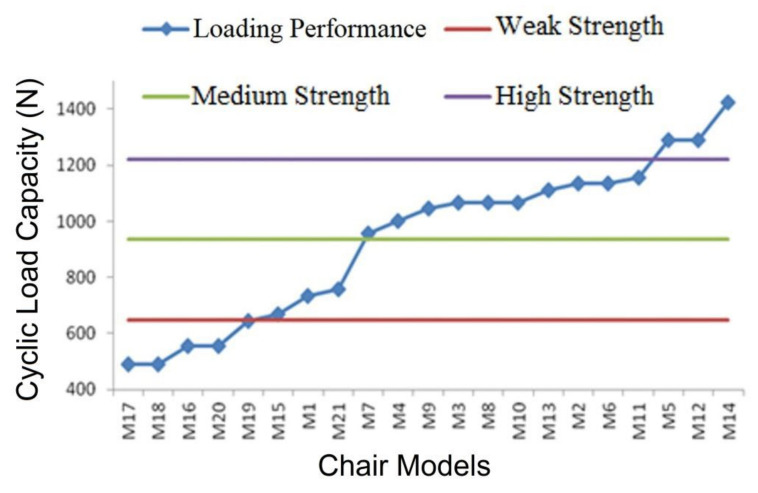
Cyclic load capacities under sidethrust direction for chair models.

The evaluation of each chair types for the classification performed for sidethrust load capacity is presented in Figure 9.

According to Figure 9, 8 chair models out of the 21, or 38%, that were tested came below the medium-strength requirements and, while being manufactured for domestic usage, were considered incapable of meeting the usage requirements. A total of 5 chair models (M17, M18, M16, M20, and M19) were categorized as having insufficient strength, 3 (M15, M1, and M21) as having weak strength, 10 (M7, M4, M9, M3, M8, M10, M13, M2, M6, and M11) as having medium strength, and 3 as having high strength. It may be said that the three high-strength models (M5, M12, and M14) were substantially stronger than required for domestic usage conditions, while the eight remaining medium-strength models still need serious strength improvement adjustments. In this group, there were 10 different varieties that may be used internally under sidethrust load capacity.

### 3.5. General Evaluation of the Tested Chair Performances

The deformation characteristics observed as a result of the performance tests and the deficiencies generally observed as a result of the strength values obtained can be summarized below:

▪ Use of defective materials;
▪ Designs with inappropriate cross-section geometry and dimensions;
▪ Constructional and application errors at the joining points;
▪ Dimensional and formal non-conformity of corner support elements;
▪ Insufficiency and inappropriateness of demountable fasteners;
▪ Not using side, front, and back intermediate rails in chair design.

According to the results of the performance tests, the critical points in the furniture frame systems are joints. In other words, the strength of the joints represents the strength of the whole system. Accordingly, it may be possible to obtain stronger furniture systems with strong joints. Factors such as suitable tenon dimensions and choosing the appropriate adhesive type and applying it in sufficient quantity and appropriately were found to be effective in the joining of the glued chair models tested in the study. In RTA chair models, factors such as the number of fasteners, the diameter of the fasteners, and the effective length of the fasteners were effective in the strength of the joints. In general, the RTA chair models tested in this study performed poorer than the glued chair models.

In the experiments, chairs without stretchers performed much worse than chairs with stretchers. It has been observed that the stretcher in the side frame affects the usage performances positively in front-to-back and back-to-front loading models. It has been observed that the stretchers, which are not widely used but located in the front and back frames of a few models, make significant contributions to the sidethrust loading performance. In the study, it is thought that the lack of stretchers, especially in RTA chairs, causes these chair models to show poor performance.

The overall evaluation of the tested chair models is shown in Table 9.

Considering the general evaluation of the performance of the chair models tested within the scope of the study; it is understood that six chair models (M8, M10, M11, M12, M13, and M14) were successful in all loading direction, six chair models (M1, M15, M18, M19, M20, andM21) did not pass any test (generally RTA chair models), and the remaining nine chair models (M2, M3, M4, M5, M6, M7, M9, M16, and M17) needed strength-improving optimizations for the loading in some directions.

According to Table 9, improvements to increase front-to-back load capacities of M3 and M9 chair models may make these chairs suitable for domestic usage. However, increasing the load capacities for M2, M5, M6, and M7 chair models, both front-to-back and back-to-front, for M4, M16, and M17 chair models, it is necessary to increase the load capacities both from the back-to-front and sidethrust direction. In general, it can be said that RTA chair models (M15, M18, M19, M20, and M21) fail in all tests, except for the M16 and M17. However, it is clear that the M16 and M17 chair models need significant improvements.

## 4. Conclusions

The chairs used in this study were procured as models from Turkish chair manufacturing companies, and their cyclic load capacities in various loading directions were identified and categorized according to their strength. For this reason, the manufacturers effective in the place where chair production was centered provided the chair models which were included in this study.

The creation of an important database with a significant number of performance test results for each chair evaluated was the study’s most noteworthy finding. For front-to-back loads, chairs performed between 845 and 2802 N, for back-to-front loads, between 445 and 1352 N, and for sidethrust loads, between 489 and 1423 N. According to the test results, there appear to be significant differences in the strengths of the various chair models. While some models are inadequate for domestic use, some models have been found to have unnecessarily excessive strength values. This situation clearly revealed inconsistency among the manufacturing companies. It is thought that this inconsistent quality (strength) level is due to the lack of R&D culture in the manufacturing companies, not allocating enough budget to R&D, not knowing the performance test methods, and not applying product engineering.

By using a variety of statistical techniques, the cyclic load capacity values acquired from the chair models are categorized according to strength. For chair models manufactured in Türkiye, as a result, acceptable design loads have been attained. For the front-to-back cyclic load capacities of chairs, it was determined that those between 932 and 1449 N are considered to be weak-strength, those between 1450 and 1968 N are considered to be medium-strength (suitable for domestic use), and those between 1968 N and above are considered to be high-strength (suitable for hotel lobbies, restaurants, libraries, etc.). In the case of back-to-front cyclic load capacities, it was determined that those between 625 and 895 N are considered to be weak-strength, those between 896 and 1167 N are considered to be medium-strength, and those between 1168 N and above are considered to be high-strength. The cyclic load capacity values for sidethrust loads are as follows: weak-strength is defined as 649–934 N, medium-strength is defined as 935–1221 N, and high-strength is defined as 1222 N and above. When the classification made in this study was compared with the acceptable design loads determined in the ALA (American Library Association) specification, which is used to evaluate chair performance in many countries of the USA and Europe, it was understood that there was no significant difference between the acceptable design loads. In front-to-back loading, the acceptable design load given in the ALA for domestic usage chairs is 1335 N, while the value found for domestic chairs in the classification in this study is 1450 N. While the recommended design load in ALA for back-to-front loading is 1001 N for domestic use, it was obtained as 896 N in the classification of this study. In case of sidethrust loading, while the recommended design load in ALA for domestic use is 890 N, the recommended value for domestic use in this study is 935 N. Accordingly, the classification developed in this study was found to be highly consistent with the values in a valid specification accepted around the world.

It is understood from the literature that the relationships between the dimensions of the furniture and the user’s anthropometric data are important in terms of ergonomics, strength, and functionality, and that the body weight of the users has a direct impact on the strength, especially in furniture used in public spaces [13,14,15]. Accordingly, it is of great importance to have acceptable design loads according to where a piece of furniture will be used and which kinds of service loads it will be exposed to, even during the design process. In this context, it is clear that the strength categorization of chairs developed in this study will make significant contributions to the furniture industry.

Prior to mass production, manufacturers should design and optimize their chair designs in terms of strength and failure modes; thus, it is critical to convert the values from this classification study into a national standard. The standardization, transmission, and display of such data to companies working for them would help them produce furniture of higher caliber, improving the quality of life for consumers in the process. Additionally, economic benefits will thereby be experienced by producers.

## Figures and Tables

**Table 1:** Results of the single sample K-S (Kolmogorov-Smirnov) test.

Loading Direction	_“_	Front- to-Back	Back-to-Front	Sidethrust
Number of Chairs		21	21	21
Normal parameters ^ab^	Average	1449.8076	895.9543	935.1438
	Std. Deviation Absolute	517.7155 0.160	271.21339	285.71450
Extreme			0.146	0.174
Differences	Positive	0.160	0.146	0.115
	Negative	−0.121	−0.139	−0.174
Kolmogorov–Smirnov Z		0.732	0.668	0.797
Probability		0.658	0.764	0.549

^a^: Test distribution is normal ^b^: Calculated from data.

**Table 2:** Some physical and mechanical properties of wood.

Turkish Beech	Minimum Value	Maximum Value	Mean Value	COV (%)
Moisture content (%)	7.59	9.32	8.45	6.99
Density (r ≈ 8%) (g/cm^3^)	0.64	0.73	0.68	8.12
Tension strength parallel to grain (N/mm^2^)	98.32	121.84	110.08	6.24
Compression strength parallel to grain (N/mm^2^)	55.68	65.49	60.58	5.22
Shear strength (N/mm^2^)	15.12	17.65	16.38	3.26
Bending strength (N/mm^2^)	109.00	152.00	130.00	11.71
Modulus of elasticity (N/mm^2^)	11063	15082	13072.5	12.23

COV: Variation coefficient.

**Table 3:** Statistical values for front-to-back load capacity tests.

Mean (N)	Standard Deviation (SD)	COV (%)	Mean + SD (N)	Mean − SD (N)
1449.8	517.7	35.7	1967.5	932.1

**Table 4:** Classification for front-to-back load capacity results of chair models.

Loading Direction	Inadequate	Weak	Medium	High
Front-to-back	≤931 N	932–1449 N	1450–1968 N	≥1969 N

**Table 5:** Statistical values for back-to-front load capacity tests.

Mean (N)	SD	COV (%)	Mean + SD (N)	Mean − SD (N)
895.9	271.2	30.2	1167.1	624.7

**Table 6:** Classification for back-to-front load capacity results of chair models.

Loading Direction	Inadequate	Weak	Medium	High
Back-to-Front	≤624 N	625–895 N	896–1167 N	≥1168 N

**Table 7:** Statistical values for sidethrust load capacity tests.

Mean (N)	SD	COV (%)	Mean + SD (N)	Mean − SD (N)
935.1	285.7	30.5	1220.8	649.4

**Table 8:** Classification for sidethrust load capacity results of chair models.

Loading Direction	Inadequate	Weak	Medium	High
Sidethrust	≤648 N	649–934 N	935–1221 N	≥1222 N

**Table 9:** Overall loading performances of the tested chair models.

Result	Chair Model	Front-to-Back	Back-to-Front	Sidethrust
Suitable for domestic use	M8	Passed	Passed	Passed
	M10	Passed	Passed	Passed
	M11	Passed	Passed	Passed
	M12	Passed	Passed	Passed
	M13	Passed	Passed	Passed
	M14	Passed	Passed	Passed
Needs to be improved	M2	Failed	Failed	Passed
	M3	Failed	Passed	Passed
	M4	Passed	Failed	Failed
	M5	Failed	Failed	Passed
	M6	Failed	Failed	Passed
	M7	Failed	Failed	Passed
	M9	Failed	Passed	Passed
	M16	Passed	Failed	Failed
	M17	Passed	Failed	Failed
Not suitable for use	M1	Failed	Failed	Failed
	M15	Failed	Failed	Failed
	M18	Failed	Failed	Failed
	M19	Failed	Failed	Failed
	M20	Failed	Failed	Failed
	M21	Failed	Failed	Failed

## Data Availability

The data used to support the findings of this study are available from the corresponding author upon request.
